# Supplementary material for: Large-Scale Production of Anti-RNase A VHH Expressed in pyrG Auxotrophic Aspergillus oryzae
Source: Curr Issues Mol Biol. 2023 May 31;45(6):4778–95. doi: 10.3390/cimb45060304 (PMC10297652; doi:10.3390/cimb45060304)
Supplement: Supplementary file 1 [file cimb-45-00304-s001.zip › cimb-2400462-supplementary.pdf]

Table S1: Comparison of heterologous expression systems in the field of VHH production

Expressed VHH	Heterologous expression system	The yield for shake flask (mg/L)	The yield of fermenter (g/L)
Anti-MUC1 VHH [36]	*E. coli* BL21 (DE3)	42-70	-
EGFR-specific VHH [37]	*E. coli* SHuffle® T7 and BL21 (DE3)	Up to 200	-
MBP-fusion VHH [38]	*E. coli* HM140	12	-
VHH against azo dyes [40]	*S. cerevisiae* VWK18gal1^{-}	0.07-0.12	0.463 and 0.608
VHH against azo-dye RR6 [65]	*S. cerevisiae* SU51	~ 100	-
Anti-EGFR VIII VHH [42]	*P. pastoris* X-33	8-10	-
Anti-AahI scorpion toxin nanobody [43]	*P. pastoris* X-33	~ 17	-
Anti-CEACAM5 nanobody [66]	*P. pastoris* GS115	51.7	-
Anti-MUC1 VHH [67]	*P. pastoris* GS115	10-15	-
VHH against azo dye R2 [69]	*A. awamori pyrG mutant*	up to 7.5	-
Arthromyces ramosus peroxidase gene (arp) fused VHH against R9 azo dye [70]	*A. awamori pyrG mutant*	10-30	-
sTAA-VHH and sTAA-N28-VHH against EGFR [71]	*A. oryzae niA mutant*	21 and 73.8	-
Glucoamylase fused VHH against human chorionic gonadotropin [72]	*A. oryzae* OSI1013 *leuA* mutant	155 and 610	-
Asp. VHH (in this study)	*A. oryzae* RIB40 *pyrG*(-)	44	1.4
